# Reasons and predictive factors for discontinuation of PDE-5 inhibitors despite successful intercourse in erectile dysfunction patients

**DOI:** 10.1038/ijir.2013.41

**Published:** 2013-12-05

**Authors:** S-C Kim, Y-S Lee, K-K Seo, G-W Jung, T-H Kim

**Affiliations:** 1 Department of Urology, Kwandong University MyongJi Hospital, Goyang, Gyeonggi-do, Korea; 2 Seo Kyung-Keun Urology Clinic, Cheonan, Korea; 3 Dr Cho&Lee's Urology Clinic, Seoul, Korea; 4 Smile Jung Gyung-Woo Urology Clinic, Busan, Korea; 5 Department of Urology, Chung-Ang University Hospital, Seoul, Korea

**Keywords:** discontinuation, erectile dysfunction, PDE5 inhibitor, predicting factor, reason

## Abstract

This study was aimed to identify characteristics of ED patients who discontinued PDE5i despite successful intercourse. Data were collected using a questionnaire from 34 urologic clinics regardless of the effect (success or failure) of PDE5i treatment by visiting the clinics (717), e-mail (64) or post (101) for 882 ED patients who had previously taken any kind of PDE5i on demand four or more times. Discontinuation of PDE5i was defined if the patient had never taken PDE5i for the previous 1 year despite successful intercourse. Of the 882 patients, 485 were included in the final analysis. Difference in the socio-demographic, ED- and partner-related data between the continuation and discontinuation group and factors influencing discontinuation of the PDE5i were analyzed. Among 485 respondents (mean age, 53.6), 116 (23.9%) had discontinued PDE5i use despite successful intercourse. Most common reasons for the discontinuation were ‘reluctant medication-dependent intercourse' (31.0%), ‘spontaneous recovery of erectile function without further treatment' (30.2%), and ‘high cost' (26.7%). In multiple logistic regression analysis, independent factors influencing discontinuation of the drug were cause of ED (psychogenic), short duration of ED, low education (⩽ middle school), and religion (Catholic). In partner-related compliance, only partner's religion (Catholic) was a significant factor.

## Introduction

Medication adherence is defined as the extent to which a person's medication-taking behavior coincides with medical advice.^1^ To be effective, disease-management interventions must take factors affecting medication adherence into account, including known factors that may influence and predict medication-taking behavior. This understanding could then become the cornerstone of an effective patient education program. Various demographic, medical, behavioral, economic, social and medication- or medical practice-related variables may affect the medication adherence.^1^

Oral PDE5i are a more convenient and noninvasive therapy for ED. PDE5i therapy has become the treatment of choice for men with ED and has proven to be efficacious in approximately 60–80% of unselected patients with ED.^2, 3^ Yet, ∼31–57% of these men abandon treatment despite successful intercourse because of the treatment.^4, 5, 6^

Various reasons for their drop-out despite successful intercourse using PDE5i have been reported but their demographic characteristics are unclear. Such knowledge would be valuable in prediction of those at increased risk of discontinuation, and would help in education programs that selectively target these men prior to the treatment to increase compliance with PDE5i use, thereby improving not only their quality of life^7^ but also that of their partners^8, 9, 10^ as possible.

The purpose of this study was to identify socio-demographic, ED- and partner-related characteristics of ED patients who discontinued PDE5i despite successful intercourse because of the treatment.

## Subjects and methods

### Eligibility criteria and methods of selection of participants

Each of the investigators from 34 urology clinics located nationwide reviewed his medical records and randomly selected ED patients who had taken any kind of PDE5i on demand four or more times, explained the aim of this study by phone call and asked them to participate in the survey between 1 May 2011 and 31 August 2011. Population of 882 ED patients ⩾19-years-of-age were recruited for this study.

**Figure 1:**
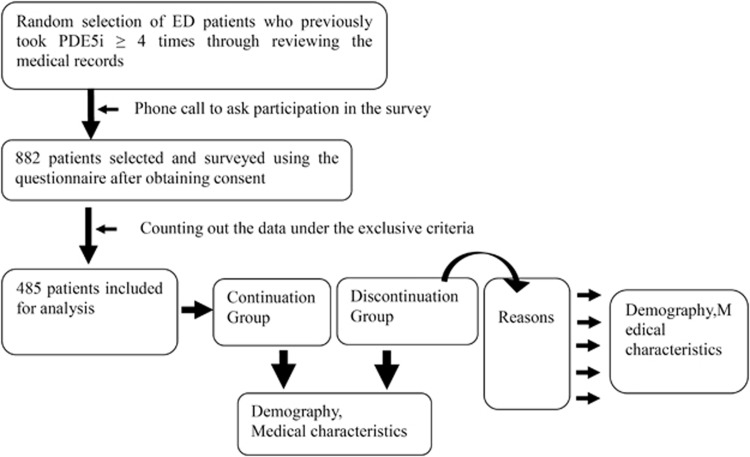
The process of patient recruitment. ED, erectile dysfunction; PDE5i, phosphodiesterase type 5 inhibitor.

A questionnaire was distributed to each of the 882 patients regardless of the effect (success or failure) of PDE5i treatment in a convenient way for the patients: by visiting the clinics (*n*=717), by e-mail (*n*=64) or by post (*n*=101). No compensation was offered to the patients to complete the survey. Prior to getting the questionnaire, the consent of each patient to participate in the survey was obtained by the physicians when they visited the physicians or by telephone. The EF was retrospectively assessed using the validated Korean version of International Index of Erectile Function-5 (IIEF-5), which is highly effective in detecting the presence and assessing the severity of ED.^11^ The Korean version of IIEF-5 revealed that the most appropriate cutoff score between ED and non-ED was 17 (sensitivity=91.3%, specificity=86.3%). The score 14–17 was classified as mild ED, 10–13 as moderate ED and less than 10 as severe ED. The recovery of EF with PDE5i was defined as IIEF-5 score ⩾18, applying the cuff-off score. Aso, ED patients whose IIEF-5 score before PDE5i treatment was ⩾18 and those with an IIEF-5 score ⩽17 after medication were excluded. The process of patient recruitment is presented in Figure 1.

Of the 882 respondents, we counted out the data of those who were not eligible for the analysis, and the data of 485 patients were included in the final analysis. The study was approved by the Institutional Review Board (IRB) of Chung-Ang University Hospital, which the principal investigator belonged to. Approval from a hospital IRB, which a principal investigator belongs to exempts another IRB approval of co-investigators' institutes in Korea.

### Questionnaire

The four-part questionnaire consisted of 40 questions (Appendix): (1) socio-demographics (seven questions regarding age, residential area, marital status and period, number of children, education, income, occupation and religion; (2) health status and lifestyle (eight questions regarding height, weight, body mass index (BMI), comorbidity, smoking, alcohol consumption, exercise and stress); (3) characteristics and treatment of ED (16 questions regarding cause and duration of ED, number of days of taking PDE5i, EF and satisfaction of intercourse (IIEF-5) before and after taking PDE5i), libido and reasons for discontinuation of PDE5i despite successful intercourse because of the treatment; (4) partner-related (nine questions regarding age, occupation, religion, comorbidity, patient's intimacy with their partner, partner's intimacy with the patient, awareness of taking PDE5i and satisfaction with the medication). The partner-related questions were directed to the patients, but not to their partners. The cause of ED was determined from the medical, social and sexual history, physical examination and the data on basic laboratory tests (fasting blood sugar, lipid profile and serum total testosterone) on the medical records. On the basis of these evaluations, the men who did not have any history nor abnormal findings related to organic ED were classified as having psychogenic ED and the others were classified as having organic ED. The number of days of taking PDE5i was estimated by summing total tablets prescribed in each clinic. We operationally defined discontinuation of PDE5i if the patient had never taken PDE5i for the past 1 year.^12^ Successful intercourse was defined as the ability to get an erection hard enough for vaginal penetration and to maintain the erection during sexual performance until ejaculation. Difference in the socio-demographic, ED- and partner-related data between the continuation and discontinuation group and the relationship between reasons for discontinuation of ED drug despite successful intercourse because of the treatment and patient characteristics were analyzed (Figure 1).

### Statistical analysis

To assess the socio-demographic characteristics that influenced compliance in the ED patients, univariate and multivariate analyses were conducted with the patient compliance (discontinuation vs continuation) as dependent variables, and with the patient socio-demographic characteristics, treatment-related characteristics, state of health and lifestyle, and partner's characteristics as independent variables. For the primary univariate analysis, the difference between the two groups (discontinuation group vs continuation group) was compared using the chi-square test or, if necessary, the Fisher's exact test, when the independent variable was categorical data and using the independent *t*-test in the case of the continuous independent variable. The Mann–Whitney U test was used for some variables without normal distribution. For the multivariate logistic analysis, the independent variables used as predictive factors were selected by considering the significant variables found by the univariate analysis and practically important factors according to expert opinion. All statistical analyses were conducted using SPSS (version 12.0). The two-sided test was used for all *P*-values (significance values) obtained by the statistical analyses.

## Results

The mean age of the 485 patients who were included in the final analysis was 53.6±11.8 years (27–87 years). Demographic characteristics of the patients are shown in Table 1 and characteristics of ED in Table 2. The severity of ED was mild in 47.0%, moderate in 46.2% and severe in 6.8%. Among the 485 patients, 116 (23.9%) had discontinued PDE5i during the past 1 year despite successful intercourse and 369 (76.1%) had continued the medication.

Reasons for the discontinuation was ‘unwilling to have medication-dependent intercourse' (31.0%) followed by ‘spontaneous recovery of EF without further medication' (30.2%), ‘high cost' (26.7%), ‘concern about side effects of the medication' (16.4%) and ‘emotionally not ready for the patient to have intercourse due to long absence of sexual intercourse' (12.9%) (Table 3). In age-specific analysis, spontaneous recovery of EF was the most common reason for respondents aged 30–39 years (58.8%), unwilling to have medication-dependent intercourse for respondents aged 40–49 years (63.6%) and concerns about side effects for respondents aged 70–79 years (35.7%) (Table 4). The proportion of discontinuation due to spontaneous recovery of EF was greater in the non-comorbid group (47.7%) than in the comorbid group (19.4%). The patients with ED duration of ⩾3 years showed a greater proportion (30.8%) of discontinuation due to high cost of the medicine than duration of<3 years (18.8%).

To investigate the relationship between socio-demographic characteristics and patient compliance, univariate analysis revealed significant differences in religion and education (Table 5), but not in age, height, residential area, marital status and period, number of children, income, occupation and age gap with partner. The proportion of Catholics in the discontinuation group (20.7%) was significantly greater than in the continuation group (9.8%) (*P*=0.015). Education showed a significant difference at the 95% confidence level. When the education was categorized into middle school graduate or below and high school graduate or above, the proportion of discontinuation was significantly greater in the former (12.9%) than the latter (7.1%) (*P*=0.049).

Among eight questions related to the state of health status and lifestyle, mean weight (kg) (69.37±8.95) and mean BMI (23.99±2.60) of the discontinuation group were significantly lower than those of the continuation groups (71.93±8.55 and 24.60±2.38, respectively) (*P*=0.006, 0.019) (Table 5). BMI ⩾23 was found significantly more often in the continuation group (85.3%) than in the discontinuation group (72.1%) (*P*=0.002).

Among the characteristics and treatment of ED, cause of ED, duration of ED symptoms, total period of the medication and total number of prescribed tablets showed significant relationship to the patient compliance. The proportion of the patients with psychogenic ED in the discontinuation group (47.4%) was significantly greater than in the continuation group (32.8%) (*P*=0.004). The mean duration of ED symptoms in the discontinuation group (4.22 years) was significantly shorter than in the continuation group (5.13 years) (*P*=0.026) (Table 5). However, there was no statistically significant difference in sexual libido and IIEF-5 scores before and after taking PDE5i between the two groups.

In multiple logistic regression analysis, independent factors influencing discontinuation of the drug were cause of ED (psychogenic), short duration of ED symptom, education (⩽middle-school) and religion (Catholic). However, weight and BMI, which showed significant differences between the continuation and discontinuation group in the univariate analysis were not independent factors influencing discontinuation of the drug (Table 6).

Among the partner-related characteristics, the proportion of patients whose partner was Catholic in the discontinuation group (25.6%) was significantly greater than in the continuation group (12.2%) (*P*=0.009). However, no other factors showed significant differences between the two groups.

## Discussion

The patients included in this study were those who took any kind of PDE5is four times or more and were able to have successful intercourse because of the treatment. We did not investigate differences in the erection effect among different kinds of PDE5i. All the PDE5is available in the current market show erection effect by the same action mechanism even though pharmacokinetics and pharmacodynamic issues are a bit different. Contemporarily, it is not possible to conclude with sufficient evidence that changing to a different PDE5i contributes to an improved erection, even though there are published studies in this respect.^13^

The presently-observed high discontinuation rate (23.9%) of PDE5i treatment despite successful intercourse because of the treatment, which is comparable to another study,^4^ suggests that successful intercourse is not the only factor involved in successful treatment and further illustrates that the need for treatment of ED is modulated by numerous outside factors. The side effects of all PDE5is available in the markets are principally the same, except for some side effects due to different tissue distribution patterns of the phosphodiesterase system.^2^ In the overwhelming majority, the drug-related adverse events resulted in only 1–2% of early drop-outs in the clinical trials.

The main reasons for the discontinuation of PED5i treatment were not different from those of other reports,^4, 5, 6^ although they were somewhat different in order of frequency. Lack of opportunity or desire for sexual intercourse (45%),^4^ effect below expectations (42.3%), high cost (37.8%)^5^ or shortcomings in the partners' or patients' emotional readiness for the restoration of sexual life after long-term abstinence (37.0%)^6^ were the leading causes of discontinuation. The higher rate (30.2%) of spontaneous recovery of EF without further treatment for the reason of discontinuation in this study, compared with other reports^5, 6^ may be attributable to majority of the participants (93.2%) were mild to moderate ED patients. Spontaneous recovery of EF and unwillingness to have medication-dependent intercourse were the most common reasons for respondents aged 30–49 years, which may be attributed to their having less impaired erectile tissue and their personal view of themselves as still being young. On the other hand, the most common reason for respondents in their 70's who were relatively unhealthier and had more co-morbidities was concern about the side effects. The cost of the treatment plays a substantial role in the medication adherence. When ED treatment was subsidized in Sweden, the cost was not an issue, but when the subsidization was withdrawn it became the most common cause (48%) for discontinuation, especially in low-income households.^14^ In this study the high cost was also a significant reason for the discontinuation, regardless of age.

Various demographic, medical, behavioral, economic, social and medication- or medical practice-related variables may affect the medication adherence. In case of ED treatment, a partner's perception and satisfaction with ED treatment also can have a substantial impact on the continuation of therapy.^15^ The questionnaire for this study on PDE5i adherence covered as many of the related variables as possible, consisting of socio-demographics, health status and lifestyle, characteristics and treatment of ED and partner-related factors. While partner issues were reported to contribute to discontinuation, none of the studies investigating adherence or discontinuation cited previously reported any contact with the partners of the men taking PDE5is until the first study^16^ which asked couples for the reasons of discontinuation despite successful treatment. The study reported a different perspective of female partners on ‘partner issues' often cited as reasons for discontinuation. For a number of women, ‘partner issues' meant a range of problems from separation to alcohol abuse, lack of communication, lack of confidence or fear of failure, while few women reported a lack of interest in sexual activity. Discontinuation did not mean couples were no longer sexually active. Our study obtained the partner-related data from the patients due to difficulties in obtaining patients' consent to contact their partners. Among 9 partner-related questions (Appendix), only the proportion of Catholic religion was significantly greater in the discontinuation group than in the continuation group.

Not every man with sexual dysfunction experiences distress and unhappiness leading to a need for therapy. In the Cologne Study, when treatment need was defined as the co-occurrence of ED and dissatisfaction with one's sex life, only 6.9% of the men were determined to be in need of ED treatment, which is significantly less than the prevalence of ED (19.2%).^17^ On the other hand, some men show positive attitudes toward sex in their life. Long-term sildenafil users tend to have a positive attitude toward sex and almost all believe that ED should be treated.^18^ Age, education, and income did not significantly influence this perception. Presently, however, respondents with less education and whose professed religion was Catholic were more likely to abandon PDE5i treatment, despite its efficacy. It is possible that those with less formal education may tend to be less active in self-care.^19^ It has been hypothesized that culture and religion play an important role in sexuality.^20^ The sacred texts of five world religions (Buddhism, Christianity, Hinduism, Islam and Judaism) use a similar belief systems to set limits on sexual behavior.^21^ In Catholicism, celibacy institutionalizes the enmity with sexuality.^22^ A further consequence of asceticism is the reduction of sexuality to reproduction. In a review of sexual instinctual gratification with religious affiliation, the weekly frequency rate of sexual intercourse amounted to 3.1% with nondenominationals, 2.6% with Protestants and 2.3% with Catholics.^22^ The present study shows the proportion of Catholics in the discontinuation group (20.7%) was significantly greater than in the continuation group (9.8%), while no such significant difference was found among Buddhists and Protestants. Furthermore, the Catholic religion was the only one among the partner-related variables that was significantly different between the discontinuation and continuation group. The effect of religion on sexuality might be different depending on culture, and further investigation is needed to know the impact of religion on sexuality, particularly to explain the different impact of each religion.

As the duration of ED symptoms is longer, the degree of ED could be more severe.^23^ Men are more likely to accept that they have ED and seek treatment for their ED with increasing duration of the condition.^24^ The patients with psychogenic ED and shorter duration of ED symptoms in this study showed significantly higher rates of discontinuation. Psychogenic ED and shorter duration of ED, which is possibly less severe ED and less likely to have pressure for seeking treatment may require less frequency of drug treatment than organic and severe ED do, which makes us guess a higher possibility of the drug discontinuation in the psychogenic and shorter duration of ED. In the Massachusetts Male Aging Study,^25^ however, the rate of trouble getting and keeping an erection in patients with mild ED was much higher (50%, 63%) and satisfaction degree with sex life (3.0 on scale from 1, extremely satisfied to 5, extremely dissatisfied) was quite lower than those of men without ED (5%, 5%) (score; 1.6), although sexual activity per month of those with mild ED was similar to men without ED. In this respect, education of the patients with less severe ED prior to treatment would be beneficial to not only patients themselves^7^ but also their partners^8, 9, 10^ for improvement of quality of life by keeping better compliance. Men with high BMI and waist circumference are more prone to sexual dysfunction compared with those with normal BMI and waist circumference.^26^ In the present study, obesity was found significantly more often in the continuation group in the univariate analysis but not in the multiple logistic regression analysis. There was no statistically significant difference in sexual libido between the continuation and discontinuation group. In a study to assess the effect of low desire on the efficacy and satisfaction rate of sildenafil in the treatment of ED, no significant difference was found between the global efficacy question response and the overall satisfaction rate between patients with and without low libido.^27^

This study has some limitations. First, the sample size was relatively small. Thus, the study population might not accurately represent the underlying population. Second, the responses to partner-related questions were obtained from the patients and so may not have always reflected reality. Third, we used the modified IIEF-5 to retrospectively assess the participants' EF before and after treatment. Recall bias cannot be avoided. Fourth, these types of surveys are invariably biased as those who choose to complete the surveys are motivated, while those who do not complete the surveys fail to do so for various reasons. Fifth, the study included only Korean men. Cultural differences might have affected our results.

In conclusion, Catholic religion, low education, psychogenic ED and short duration of ED symptoms were predictive factors for the discontinuation of PDE5i despite successful intercourse in Korean men with ED. Thus, an effective education program for these men prior to the treatment would be valuable to increase the compliance with PDE5i treatment.

## Figures and Tables

**Table 1:** Characteristics of the 485 patients

	*Number (%)*	
	*Patients*	*Partners*
*Age, years*		
20–29	11 (2.3)	5 (1.0)
30–39	51 (10.5)	58 (12.0)
40–49	113 (23.3)	124 (25.6)
50–59	156 (32.2)	133 (27.4)
60–69	103 (21.2)	73 (15.1)
⩾70	47 (9.7)	17 (3.5)
No answer	4 (0.8)	75 (15.5)
		
*Education*		
No education	1 (0.2)	3 (0.6)
Elementary	11 (2.3)	17 (3.5)
Middle school	29 (6.0)	47 (9.7)
High school	162 (33.4)	174 (35.9)
University	238 (49.1)	162 (33.4)
Post-graduate	42 (8.7)	12 (2.5)
No answer	2 (0.4)	70 (14.4)
		
*Occupation*		
White collar	153 (31.5)	59 (12.2)
Blue collar	102 (21.0)	10 (2.1)
Self-employed/Service	155 (32.0)	66 (13.6)
Others/No job	75 (15.5)	280 (57.7)
No answer	0 (0.0)	70 (14.4)
		
*Religion*		
Protestant	95 (19.6)	94 (19.4)
Catholic	60 (12.4)	58 (12.0)
Buddhist	131 (27.0)	126 (26.0)
Others	199 (41.0)	96 (19.8)
No answer	0 (0.0)	111 (22.9)
		
*Co-morbidities*		
Diabetes mellitus	58 (12.0)	27 (5.6)
Hypertension	102 (21.0)	54 (11.1)
Dyslipidemia	39 (8.0)	15 (3.1)
Obesity	46 (9.5)	27 (5.6)
Coronary artery disease	14 (2.9)	3 (0.6)
Benign prostatic hyperplasia	119 (24.5)	7 (1.4)
Arthritis	13 (2.7)	19 (3.9)
Herniated nucleus pulposus	17 (3.5)	15 (3.1)
Digestive disorder	25 (5.2)	25 (5.2)
Others	43 (8.9)	25 (5.2)
		
*Residential area*		
Urban	446 (92.0)	
Rural	37 (7.6)	
No answer	2 (0.4)	
		
*Marital status*		
Marriage/Cohabit	416 (85.8)	
Bereavement	11 (2.3)	
Divorce	14 (2.9)	
Separation	13 (2.7)	
Bachelor	25 (5.2)	
Others	6 (1.2)	
		
*Marital period* (*years)*		
<1	4 (0.8)	
1–3	12 (2.5)	
4–9	22 (4.5)	
⩾10	371 (76.5)	
No answer	76 (15.7)	
		
*Monthly income*		
<1 million Korean Won (KRW)	20 (4.1)	
1-2 million KRW	50 (10.3)	
2-3 million KRW	91 (18.8)	
3-4 million KRW	105 (21.6)	
4-5 million KRW	76 (15.7)	
⩾5 million KRW	142 (29.3)	
No answer	1 (0.2)	
		
*Smoking*		
No	310 (63.9)	
Yes (pack)	175 (36.1)	
<0.5	34 (7.0)	
0.5–1	67 (13.8)	
1 -<2	71(14.7)	
⩾2	2 (0.4)	
No answer	1 (0.2)	
		
*Alcohol drinking*		
No	120 (24.7)	
Yes	365 (75.3)	
5–7 days per week	33 (6.8)	
3-4 days per week	97 (20.0)	
1-2 days per week	141 (29.1)	
1-2 days per month	72 (14.8)	
<1 day per month	20 (4.1)	
No answer	2 (0.4)	
		
*Exercise*		
No	71 (14.6)	
1 day per week	95 (19.6)	
2 days per week	68 (14.0)	
3 days per week	104 (21.4)	
4 days per week	60 (12.4)	
⩾5 days per week	83 (17.1)	
No answer	4 (0.8)	
		
*Stress*		
Hardly	68 (14.0)	
Somewhat	222 (45.8)	
Highly	167 (34.4)	
Very highly	26 (5.4)	
No answer	2 (0.4)	

**Table 2:** Characteristics of erectile dysfunction

	*Number (%)*
*Duration of ED symptom (years)*	
<5	276 (56.9)
5–9	125 (25.8)
10–14	48 (9.9)
⩾15	12 (2.5)
Don't know/No answer	24 (4.9)
	
*Libido*	
Low	21 (4.3)
Medium	311 (64.1)
High	153 (31.5)
	
*Cause of ED*	
Psychogenic	176 (36.3)
Organic	309 (63.7)
	
*Number of medication (times)*	
4–10	124 (25.6)
⩾10	361 (74.4)

*Severity of ED*	*Before taking PDE5i*	*After taking PDE5i*
Mild (14–17^a^)	228 (47.0)	0 (0.0)
Moderate (10–13^a^)	224 (46.2)	0 (0.0)
Severe (⩽9^a^)	33 (6.8)	0 (0.0)

Abbreviations: ED, erectile dysfunction; PDE5i, phosphodiesterase type 5 inhibitor.

a Korean version of International Index of Erectile Function (IIEF)-5 score.

**Table 3:** Reasons for discontinuation of PDE5i - multiple choice responses

	*Number*	*%*
Unwillingness to depend on the medicine for sexual intercourse	36	31.0
Spontaneous recovery of erectile function without PDE5i	35	30.2
High cost of the medicine	31	26.7
Concerns about side effects of the medicine	19	16.4
Patients' lack of emotional readiness for restoration of sexual activity after long-term abstinence	15	12.9
Psychological burden of scheduled sexual intercourse	11	9.5
Unwillingness to have sexual intercourse	9	7.8
Discontinuation of the erectile dysfunction medicine because it was more important to treat other disease	7	6.0
Partner unpreparedness due to long absence of sexual intercourse	6	5.2
Others	12	5.2
Total	116	100.0

**Table 4:** Reasons for discontinuation of PDE5i in age-specific analysis

*Reasons for discontinuation*		*Age*				
	*Total*	*30's*	*40's*	*50's*	*60's*	*70's*
	*(n=116)*	*(n=17)*	*(n=22)*	*(n=35)*	*(n=27)*	*(n=14)*
	*%*	*%*	*%*	*%*	*%*	*%*
Unwillingness to depend on the medicine for sexual intercourse	31.0	23.5	63.6	20.0	25.9	21.4
Spontaneous recovery of erectile function	30.2	58.8	27.3	37.1	22.2	—
High cost of the medicine	26.7	29.4	36.4	22.9	22.2	28.6
Concerns about side effects of the medicine	16.4	23.5	18.2	8.6	11.1	35.7
Patients' lack of emotional readiness for restoration of sexual activity after long-term abstinence	12.9	5.9	13.6	17.1	7.4	21.4
Psychological burden of scheduled sexual intercourse	9.5	11.8	4.5	11.4	14.8	—
Unwillingness to have sexual intercourse	7.8	5.9	9.1	2.9	14.8	7.1
Discontinuation of the medicine because it was more important to treat other disease	6.0	11.8	9.1	—	11.1	—
Partner unpreparedness due to long absence of sexual intercourse	5.2	—	4.5	11.4	—	7.1

**Table 5:** Significantly different variables between the discontinuation and continuation group in univariate analysis

*Variables*	*Discontinuation* n *(%)*	*Continuation* n *(%)*	P*-value*
a*Religion*			
Protestant	19 (16.4)	76 (20.6)	
Catholic	24 (20.7)	36 (9.8)	0.015
Buddhist	26 (22.4)	105 (28.5)	
Others	47 (40.5)	152 (41.2)	
			
a*Education status*			
⩽ Middle school	15 (12.9)	26 (7.1)	0.049
> Middle school	101 (87.1)	341 (92.9)	
			
a*BMI (kg/m*^*2*^)			
<23	29 (27.9)	47 (14.7)	0.002
⩾23	75 (72.1)	273 (85.3)	
			
a*Cause of ED*			
Psychogenic	55 (47.4)	121 (32.8)	0.004
Organic	61 (52.6)	248 (67.2)	

*Variables*	*Discontinuation mean±s.d. (n)*	*Continuation mean±s.d. (n)*	P*-value*
bWeight (kg)	69.37±8.95 (116)	71.93±8.55 (368)	0.006
bBMI (kg/m^2^)	23.99±2.60 (116)	24.60±2.38 (368)	0.019
cDuration of ED (year)	4.22±3.33 (110)	5.13±3.87 (351)	0.026
cMedication period (month)	15.71±24.57 (16)	28.44±24.03 (369)	<0.001
cNumber of prescribed tablets	19.13±22.85 (16)	53.31±63.16 (369)	<0.001

Abbreviations: BMI, body mass index; ED, erectile dysfunction.

a Chi-square test.

b Independent Sample *t*-test.

c Mann–Whitney U test.

**Table 6:** Multiple Logistic Regression analysis of associated factors with discontinuation of medication

*Factor*	*B*	*s.e.*	*OR*	*95% CI*	P*-value*
Age	0.02	0.01	1.02	0.99–1.04	0.15
Number of co-morbidity	−0.01	0.09	0.99	0.84–1.17	0.91
Duration of ED symptom	−0.08	0.04	0.93	0.86–0.99	0.03^a^
BMI (kg/m^2^)	−0.08	0.05	0.92	0.84–1.01	0.09
Stress^b^	0.01	0.16	1.01	0.73–1.37	0.98
Exercise^c^	−0.02	0.08	0.98	0.84–1.13	0.76
					
*Education*					
⩽ Middle School			1		
>Middle School	−0.74	0.37	0.48	0.23–0.99	0.05^a^
					
*Marital Status*					
Living without partner			1		
Living with partner	0.09	0.33	1.09	0.57–2.10	0.79
					
*Religion*					
Non-Catholic			1		
Catholic	0.84	0.31	2.31	1.26–4.24	0.01^a^
					
*Smoking status*					
Non-smoker			1		
Smoker	−0.02	0.26	0.98	0.59–1.63	0.94
					
*Drinking status*					
Non-Drinker			1		
Drinker	−0.07	0.27	0.93	0.55–1.57	0.78
					
*ED Cause*					
Organic			1		
Psychological	0.63	0.26	1.88	1.12–3.13	0.02^a^

Abbreviations: s.e., standard error; OR, odds ratio; CI, confidence interval.

a *P*<0.05 by multiple logistic regression.

b Score Range [1: hardly to 4: very highly].

c Score Range [1: never to 6: more than 5 days per week].
